# Overexpression of *SlALC* Increases Drought and Salt Tolerance and Affects Fruit Dehiscence in Tomatoes

**DOI:** 10.3390/ijms25179433

**Published:** 2024-08-30

**Authors:** Zihan Gao, Yuqing Tu, Changguang Liao, Pengyu Guo, Yanling Tian, Ying Zhou, Qiaoli Xie, Guoping Chen, Zongli Hu

**Affiliations:** Laboratory of Molecular Biology of Tomato, Bioengineering College, Chongqing University, Chongqing 400030, China; gzhfilm@163.com (Z.G.); tuyuqin1998@163.com (Y.T.); changguangliao@163.com (C.L.); guopengyucqu@163.com (P.G.); tianna0816@163.com (Y.T.); 15198904590@163.com (Y.Z.); qiaolixie@cqu.edu.cn (Q.X.)

**Keywords:** *SlALC*, abiotic stress, fruit dehiscence, lignification, tomato

## Abstract

The bHLH transcription factors are important plant regulators against abiotic stress and involved in plant growth and development. In this study, *SlALC*, a gene coding for a prototypical DNA-binding protein in the bHLH family, was isolated, and *SlALC*-overexpression tomato (*SlALC*-OE) plants were generated by Agrobacterium-mediated genetic transformation. *SlALC* transgenic lines manifested higher osmotic stress tolerance than the wild-type plants, estimated by higher relative water content and lower water loss rate, higher chlorophyll, reducing sugar, starch, proline, soluble protein contents, antioxidant enzyme activities, and lower MDA and reactive oxygen species contents in the leaves. In *SlALC*-OE lines, there were more significant alterations in the expression of genes associated with stress. Furthermore, *SlALC*-OE fruits were more vulnerable to dehiscence, with higher water content, reduced lignin content, SOD/POD/PAL enzyme activity, and lower phenolic compound concentrations, all of which corresponded to decreased expression of lignin biosynthetic genes. Moreover, the dual luciferase reporter test revealed that SlTAGL1 inhibits *SlALC* expression. This study revealed that *SlALC* may play a role in controlling plant tolerance to drought and salt stress, as well as fruit lignification, which influences fruit dehiscence. The findings of this study have established a foundation for tomato tolerance breeding and fruit quality improvement.

## 1. Introduction

The bHLH family members contain two conserved domains: the N-terminal DNA-binding domain and the C-terminal HLH (helix-loop-helix) domain [1]. It has been demonstrated that bHLH transcription factors exert control over plant tolerance by regulating the transcription of related genes via binding to specific cis-acting motifs in the promoter of these genes. This regulatory mechanism influences plant development and metabolic activities, including photosynthesis, shade avoidance, and production of secondary metabolites [2]. The bHLH class of transcription factors has a significant role in regulating the response of plants to drought. For instance, overexpression of the maize *ZmPIF3* gene in rice plants improves their ability to withstand drought conditions by controlling the expression of stress-related genes, including *Rab16D*, *DREB2A*, *Rab21*, *BZ8*, and *P5CS* [3]. The upregulation of *MfPIF1*, a gene from *Selaginella tamariscina* (the resurrection grass), led to enhanced tolerance to drought via controlling the opening of stomata in *Arabidopsis thaliana* [4]. Ectopic overexpression of the populus *PebHLH35* in *Arabidopsis* improved plant tolerance to drought stress by regulating stomatal density and aperture and was a positive regulator of the stress response [5]. Rice (*Oryza sativa*) *OsbHLH148* regulated the *OsJAZ* gene involved in the jasmonate signaling pathway and interacted with the ZIM domain protein OsJAZ1 to regulate plant response to drought [6].

The bHLH transcription factors are also important in regulating plant responses to salt stress. The overexpression of the wheat *TabHLH39* gene in *Arabidopsis* resulted in a notable enhancement of plant tolerance to salt stress through the regulation of sugar signaling and metabolism, leaf senescence, and cellular osmotic pressure [7]. Additional research has demonstrated that exposure to high levels of salt triggers the activation of *CabHLH035* in pepper plants. Furthermore, when *CabHLH035* was artificially introduced into *Arabidopsis*, it enhanced the plant’s ability to withstand salt stress by influencing the balance of sodium and potassium ions and promoting the production of proline [8]. Overexpression of *MfbHLH38* in *Arabidopsis* improved salt stress tolerance by increasing water retention capacity and reducing stress-induced oxidative damage [9]. Moreover, the overexpression of *EcbHLH57* in tobacco has the capacity to accelerate root growth and greatly increase the salt tolerance of tobacco plants [10].

Fruit size and shape [11], fruit growth rate [12], water content [13], mechanical properties of the pericarp [14], and expression of genes related to fruit dehiscence [15,16,17] are currently recognized as factors affecting fruit dehiscence, along with external factors such as temperature, humidity [18], and cultural practices [19]. Studies have demonstrated that bHLH family transcription factors are crucial in the process of fruit dehiscence in plants. In peach (*Prunus persica*), a *bHLH* gene controlled the development of endocarp margins during core hardening in crack-resistant and crack-prone cultivars [20]. The bHLH protein IND in *Arabidopsis* was essential for angiosperm dehiscence and has an impact on seed dispersal [21]. An analysis was conducted on the expression of the *SPT/ALC* gene in poppy, revealing that members of this lineage exhibit a high degree of functional conservation at the carpel margin and dehiscence region [22]. The current study of the effect of bHLH transcription factors on fruit dehiscence is mainly focused on the *ALC/SPT* system, which is involved in the process of fruit lignification and consequently fruit dehiscence.

In this study, a *bHLH* gene, *SlALC*, was isolated from tomato (*Solanum lycopersicum* L.), and its overexpression in tomato plants improved their drought and salt tolerance. Various stress-related physiological indicators also demonstrated the tolerance of *SlALC*-OE plants. Furthermore, *SlALC*-OE fruits were found to be more susceptible to dehiscence after rainfall, and fruit dehiscence-related indicators such as water content, lignin content, and oxidative enzyme activity showed a decreasing trend, while the expression of genes related to fruit lignification was also consistently downregulated. Subsequent investigations revealed that tomato *SlTAGL1* has the ability to exert a negative influence on the promoter activity of *SlALC*. This indicates that SlTAGL1 impacts the process of fruit dehiscence by controlling the mRNA level of *SlALC*. This study focuses on the involvement of *SlALC* in regulating plant responses to drought and salt stress and affecting the mechanism of tomato fruit dehiscence. 

## 2. Results

### 2.1. Bioinformatics Analysis, Subcellular Localization, and Expression Pattern of SlALC

**Figure 1:**
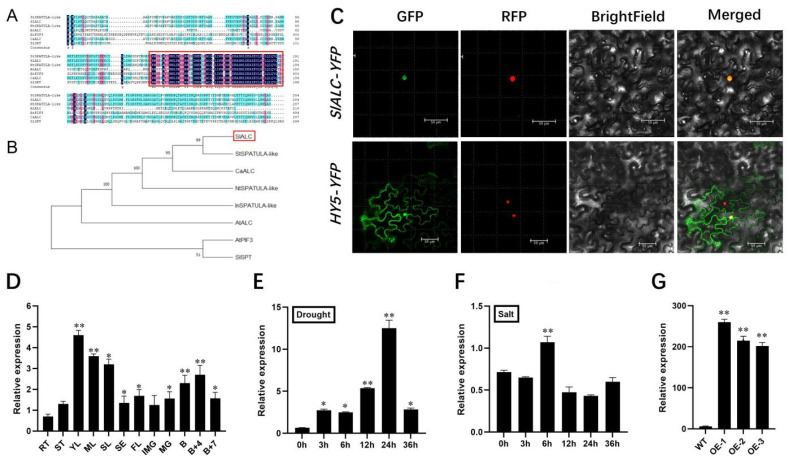
Bioinformatics analysis, subcellular localization, and expression pattern of *SlALC*. (**A**) Homology analysis of SlALC; the black box indicates the PIFs domain. (**B**) SlALC phylogenetic tree. The accession numbers are SlALC (NP_001361317, highlighted in red box), StSPATULA-like (XP_049391100), CaALC (NP_001361321), NtSPATULA-like (XP_009627217.1), InSPATULA-like (XP_019182870), AtALC (AT5G67110), AtPIF3 (AT1G09530), and SlSPT (NP_001361318.1). (**C**) Subcellular localization assay of SlALC protein. GFP: green fluorescent protein; RFP: red fluorescent protein. Red fluorescent protein is used to locate the nucleus. Scale bar = 50 µm. (**D**) Quantitative RT–PCR analysis of the expression of the *SlALC* gene in roots (RT), stems (ST), young leaves (YL), mature leaves (ML), senescent leaves (SL), sepals (SE), flowers (FL), and fruits (pericarp) at immature green (IMG), mature green (MG), breaker (B), B+ 4 and B + 7 stages. (**E**,**F**) Expression patterns of *SlALC* in leaves under the dehydration and salt treatments. (**G**) Relative expression of *SlALC* in leaves of *SlALC*-OE T0 plants. Data are means ± SD of three biological replicates. Statistically significant differences were determined using Student’s *t* test (* *p* < 0.05, ** *p* < 0.01).

The gene information for *SlALC* (*Solyc04g078690*) was analyzed using the National Center of Biotechnology Information (NCBI). It is located on tomato chromosome 4, with an mRNA length of 1278 bp. *SlALC* codes for an unstable, lipophilic protein consisting of 314 amino acids and has an isoelectric point of 5.33. Multiple protein sequence alignments showed that ALC proteins are highly conserved across species, all having PIF structural domains, and are typical bHLH proteins (Figure 1A). Phylogenetic tree analysis of SlALC homologous proteins using MEGAX showed that SlALC has the highest homology with potato (*Solanum tuberosum*) StSPATUA-like as well as chili pepper (*Capsicum frutescens*) CaALC (96.82% and 89.47% homology, respectively), but the function of the two proteins has not been reported at present (Figure 1B). Moreover, the promoter analysis revealed that the promoter of the *SlALC* gene primarily consists of three types of elements: phytohormones, light, and stress response (Table S2). To investigate the subcellular localization of SlALC protein, a 35S::SlALC-GFP fusion vector was constructed and co-injected with the nuclear localization vector for transient expression in tobacco leaves. The results displayed that the green fluorescence emitted by SlALC-GFP coincided with the red fluorescence emitted by the nuclear localization carrier, implying that SlALC is a protein predominantly localized in the nucleus (Figure 1C). In order to examine the possible role of *SlALC* in the growth and development, the expression levels of *SlALC* in various tissues and organs of tomatoes were quantitatively analyzed using qRT-PCR. The results showed that *SlALC* transcript levels were highest in young leaves, followed by mature leaves and senescent leaves and fruits, and lowest in roots (Figure 1D). Due to the presence of a stress response element in the *SlALC* promoter, we quantitatively analyzed the expression level of *SlALC* in WT tomatoes before and after drought and salt treatments (Figure 1E,F). Along with the time of drought stress, accumulation of *SlALC* mRNA gradually increased and reached maximum at 24 h, then rapidly decreased. However, the expression level of *SlALC* reached the highest point at 6 h of salt treatment, then recovered. The results indicated that *SlALC* may be involved in the response to drought and salt stress in tomatoes. 

### 2.2. Overexpression of SlALC Confers Tolerance to Mannitol and Salt at the Stages of Seed Germination and Seedling Growth

**Figure 2:**
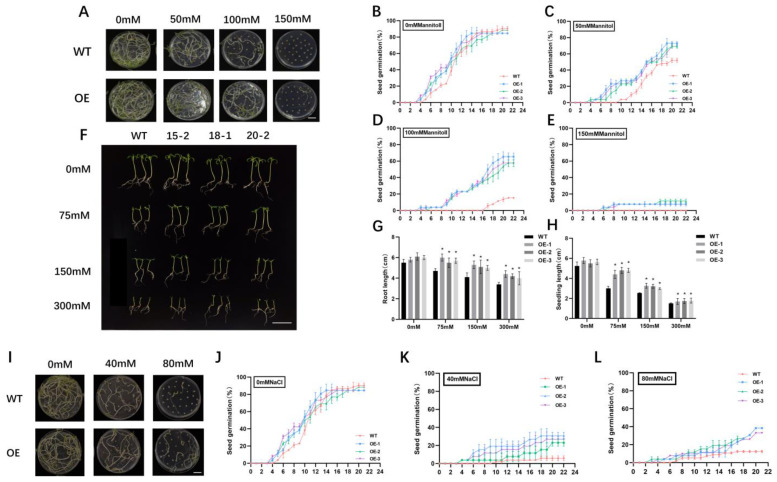
Mannitol and salt tolerance analysis of WT and transgenic seeds and seedlings. (**A**) Germination phenotype of WT and *SlALC*-OE seeds under 0, 50, 100, and 150 mM Mannitol treatments for 3 weeks. Scale bar = 1 cm. (**B**–**E**) Seed germination rates of WT and *SlALC*-OE lines under 0, 50, 100, and 150 mM Mannitol treatment, respectively. (**F**) Phenotypic map of *SlALC*-OE seedlings under 0, 75, 150, and 300 mM mannitol treatment. Scale bar = 5 cm. (**G**,**H**) Root and seedling length of WT and *SlALC*-OE plants under normal and 0, 75, 150, and 300 mM mannitol treatment, respectively. (**I**) Germination phenotype of WT and *SlALC*-OE seeds under 0, 40, and 80 mM NaCl treatments for 3 weeks. Scale bar = 1 cm. (**J**–**L**) Seed germination rates of WT and *SlALC*-OE lines under 0, 40, and 80 mM NaCl treatments, respectively. Data are means ± SD of three biological replicates. Statistically significant differences were determined using Student’s *t* test (* *p* < 0.05).

To better elucidate the biological functions of *SlALC*, we generated three independent *SlALC*-overexpression transgenic lines with dramatically higher transcript levels of *SlALC* compared with wild-type for subsequent studies (Figure 1G). First, *SlALC*-OE and WT seeds were subjected to mannitol treatment with different concentrations (0 mM, 50 mM, 100 mM, and 150 mM of mannitol) (Figure 2A). In the control group with 0 mM mannitol treatment, the germination rate of *SlALC*-OE and WT seeds was identical (Figure 2B). However, when the concentration of mannitol increased, the germination rate of *SlALC*-OE seeds was notably higher than that of the WT (Figure 2C,D). When exposed to a 150mM mannitol treatment, no seeds of WT germinated; nevertheless, a 10% germination rate was observed in *SlALC*-OE lines (Figure 2E). The results indicated that overexpression of *SlALC* in tomatoes increases the tolerance of seeds to mannitol.

Later, we carried out the mannitol treatment on seedlings of WT and overexpression lines with a gradient of four concentrations (0, 75, 150, and 300 mM of mannitol) to investigate the sensitivity of tomato seedlings to mannitol (Figure 2F). The results showed that the root and hypocotyl lengths of both WT and *SlALC*-OE seedlings were repressed under mannitol stress, but the root and hypocotyl lengths of the *SlALC*-OE seedlings were always longer than those of the WT at the same concentration (Figure 2G,H).

Simultaneously, we investigated the effect of salt stress on seed germination of the WT and *SlALC*-OE lines with different concentrations of salt solution (0 mM, 40 mM, and 80 mM of NaCl). These results showed that WT and *SlALC*-OE seeds have no difference in germination rates without NaCl treatment, yet the germination of WT and *SlALC*-OE seeds was greatly suppressed when exposed to salt stress, and the germination rates of the *SlALC*-OE lines were higher than that of WT under the same concentration (Figure 2J–L). These results indicated that overexpression of *SlALC* in tomatoes enhances the seeds’ tolerance to salt stress.

### 2.3. Overexpression of SlALC Increases Drought and Salt Tolerance of Tomato Plant

**Figure 3:**
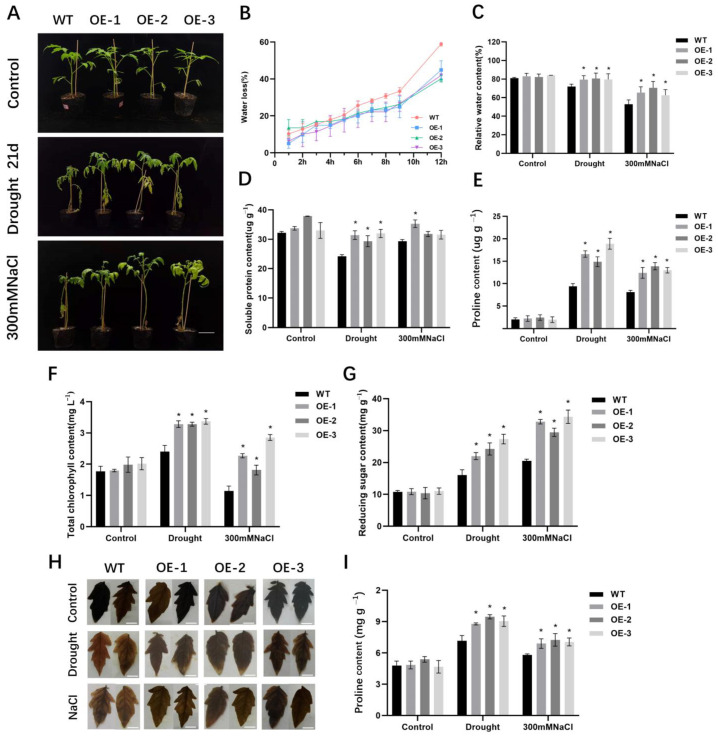
The phenotype of WT and *SlALC*-OE transgenic tomato plants under drought and salt stress. (**A**) Growth Status of WT and *SlALC*-OE under drought and salt stress. Scale bar = 10 cm. (**B**–**I**) Comparisons of water loss rate (**B**), relative water content (**C**), soluble protein content (**D**), proline content (**E**), total chlorophyll content (**F**), reducing sugar content (**G**), starch content (**I**). (**H**) KI/I2 staining. Scale bar = 1 cm. Data are means ± SD of three biological replicates. Statistically significant differences were determined using Student’s *t* test (* *p* < 0.05).

Further, the effect of drought and salt stress on the 5-week-old *SlALC*-OE and WT plants was investigated. After 21 days of drought and salt stress, wild-type plants exhibited pronounced chlorosis, wilting, and necrosis of the lower foliage, and the stem could not stand upright by itself, whereas the *SlALC*-OE plants showed slightly wilting and yellowing, and the upper leaves were still green (Figure 3A). To further understand the tolerance phenotype of *SlALC*-OE lines to drought and salt stress, some physiological parameters were analyzed. As shown in Figure 3B, the water loss rate of detached leaves of WT was significantly faster than that of *SlALC*-OE lines. After drought and salt treatments, *SlALC*-OE leaves had higher relative water content, soluble proteins, proline, chlorophyll, reducing the sugar and starch content compared with WT (Figure 3C–I). Later, the iodine-starch staining experiment was performed and demonstrated that more starch was accumulated in transgenic leaves under stresses (Figure 3H).

**Figure 4:**
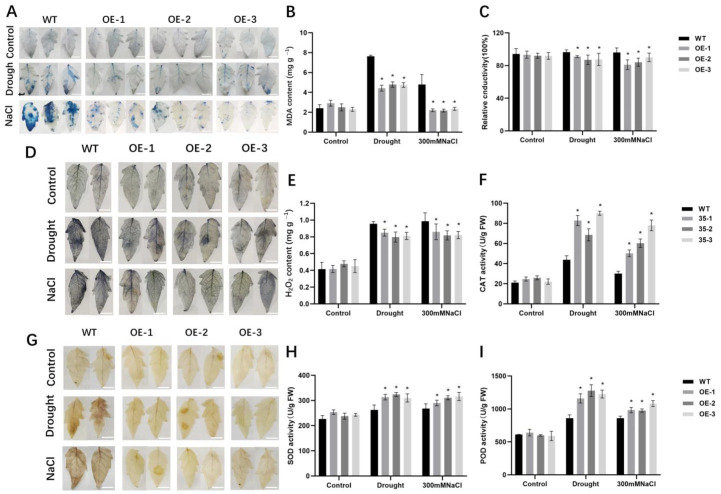
Comparison of cell damage indicators, ROS content between WT and *SlALC*-OE lines under drought and salt treatments. (**A**) Trypan blue staining, (**B**) MDA content, (**C**) relative conductivity, (**D**) NBT staining, (**E**) H_2_O_2_ content, (**F**) CAT activity, (**G**) DAB staining, (**H**) SOD activity, (**I**) POD activity. Scale bar = 1 cm. Data are means ± SD of three biological replicates. Statistically significant differences were determined using Student’s *t* test (* *p* < 0.05).

To further characterize the degree of tomato leaf damage under stress conditions, the detached leaves were stained with trypan blue. The results showed that *SlALC*-OE leaves had fewer dead cells than the WT under drought and salt stress (Figure 4A). Moreover, the MDA content and relative conductivities of the *SlALC*-OE leaves were also significantly lower than those of the WT (Figure 4B,C). These results suggested that the cells of the *SlALC*-OE plants suffered less damage under stress conditions. To assess the accumulation of reactive oxygen species (ROS) in leaves, we used 3,3-diaminobenzidine (DAB) and nitroblue tetrazolium (NBT) staining to qualitatively determine the accumulation of hydrogen peroxide (H_2_O_2_) and superoxide anion (O_2_^−^), respectively. After stress treatment, the *SlALC*-OE leaves had fewer blue and brown areas than the WT, indicating less accumulation of O_2_^−^ and H_2_O_2_ (Figure 4D,G), which were confirmed by the examination of H_2_O_2_ content in the leaves (Figure 4E).

The content of ROS in plants usually increases under stress conditions, which activates the antioxidant enzyme system in plants to eliminate ROS. We therefore investigated the enzyme activities of catalase (CAT), superoxide dismutase (SOD), and peroxidase (POD) in tomato leaves. The activities of the three enzymes in the *SlALC*-OE lines were significantly higher than those of the WT (Figure 4F,H,I), suggesting that the increased activities of antioxidant enzymes in *SlALC*-OE plants reduce the accumulation of ROS, subsequently conferring tolerance to drought and salt stress.

### 2.4. Expression Levels of Stress-Related Genes in SlALC-OE Lines under Drought and Salt Stress 

**Figure 5:**
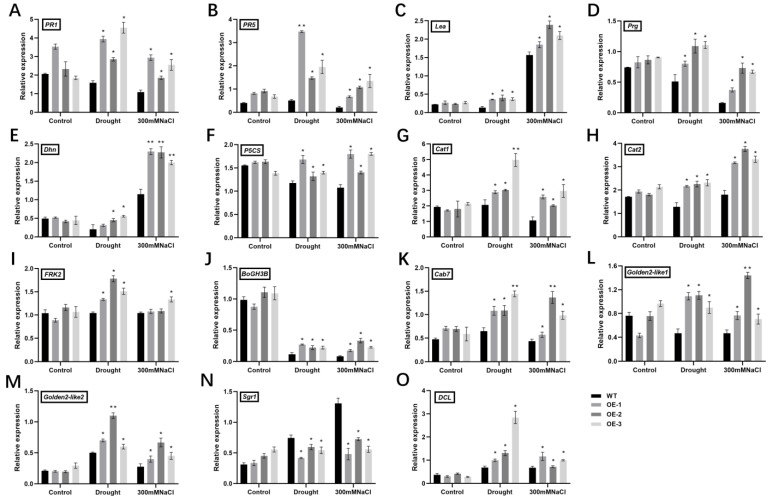
Expression levels of stress-related genes before and after drought and salt treatment in WT and *SlALC*-OE mature leaves. (**A**–**O**) qRT-PCR analysis of the expression levels of *PR1*, *PR5*, *Lea*, *Prg*, *Dhn*, *P5CS*, *Cat1*, *Cat2*, *FRK2*, *BoGH3B*, *Cab7*, *Golden2-like1*, *Golden2-like2*, *Sgr1*, *DCL*. Data are means ± SD of three biological replicates. Statistically significant differences were determined using Student’s *t* test (* *p* < 0.05, ** *p* < 0.01).

To explore the enhanced stress tolerance of *SlALC*-OE lines at the molecular level, the expression of genes involved in biotic (*PR1* and *PR5* [23]) and abiotic (*Lea*, *Prg*, *Dhn*, *P5CS* [16,24,25]) stress responses was examined. After drought and salt treatments, the mRNA abundance of these detected genes evidently increased compared with the WT (Figure 5A–F). In particular, after drought treatment, the expression of the *PR5* gene in the transgenic lines was 3.5–8.5 times higher than that of the WT (Figure 5B). The results suggested that overexpression of *SlALC* enhances stress tolerance of tomato plants via upregulating the expression of stress-related genes under stress conditions.

Based on the above experimental results indicating that *SlALC*-OE plants have strong antioxidant enzyme activities, two peroxidase genes were tested: *Cat1* and *Cat2* [26]. The results showed that the expression levels of both genes in *SlALC*-OE plants were remarkably increased after drought and salt stress (Figure 5G,H), suggesting that overexpression of *SlALC* improves the ability to scavenge hydrogen peroxide under stress, which is consistent with the DAB staining results as well as changes in hydrogen peroxide content (Figure 4G,E).

This study found higher chlorophyll, starch, and reducing sugar contents in the *SlALC*-OE lines than in the WT after stress treatment. We examined the expression of genes that positively regulate starch and sugar metabolism, *FRK2* and *BoGH3B* [27,28], and chlorophyll accumulation-related genes *Golden2-like1*, *Golden2-like2*, *Sgr1*, and *DCL* [29,30]. These results showed that the expression of *FRK2* and *BoGH3B* genes was significantly increased in the transgenic lines after drought and salt stress (Figure 5I,J), and the positively regulated genes of chlorophyll synthesis, *Golden2-like1*, *Golden2-like2*, and *DCL*, were also upregulated in the *SlALC*-OE lines (Figure 5L,M,O), while the negatively regulated gene of chlorophyll synthesis, *Sgr1*, was down-regulated in the transgenic lines (Figure 5N). These results suggested that *SlALC* may be involved in the stress response by regulating starch, sugar, and chlorophyll synthesis in tomato plants.

### 2.5. Overexpression of SlALC Gene Affects Fruit Dehiscence

**Figure 6:**
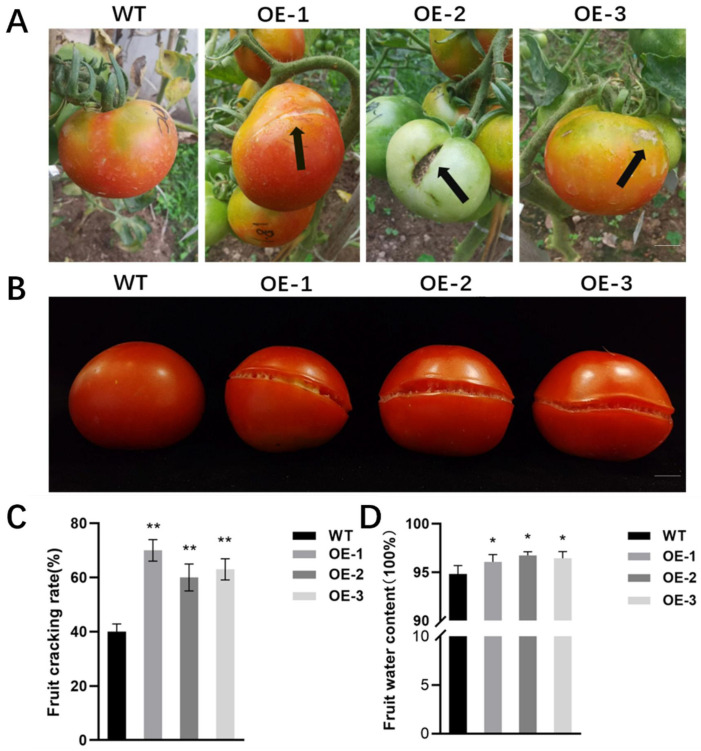
*SlALC*-OE lines with cracks in fruit. (**A**) The phenotype of cracking fruit after rain. Black arrow indicates tomato fruit cracks. (**B**) Fruit cracks in *SlALC*-OE lines compared with WT. (**C**) Fruit cracking rate. (**D**) Fruit water content. Scale bar = 1 cm. Data are means ± SD of three biological replicates. Statistically significant differences were determined using Student’s *t* test (* *p* < 0.05, ** *p* < 0.01).

After a period of artificial rainfall, more cracked fruits were observed in the *SlALC*-OE lines than in WT (Figure 6A,B); therefore, subsequently, the rate of fruit dehiscence was counted. The wild-type fruits showed a 40% cracking rate, while the transgenic lines had a 60–75% cracking rate (Figure 6C). The results suggested that exposure to rainwater caused the skin of *SlALC*-OE fruits to crack more easily. In addition, the water content of *SlALC*-OE fruits was higher than that of the WT (Figure 6D), suggesting that the higher water content might be the reason why *SlALC*-OE fruits were more susceptible to dehiscence after the rain than the WT.

**Figure 7:**
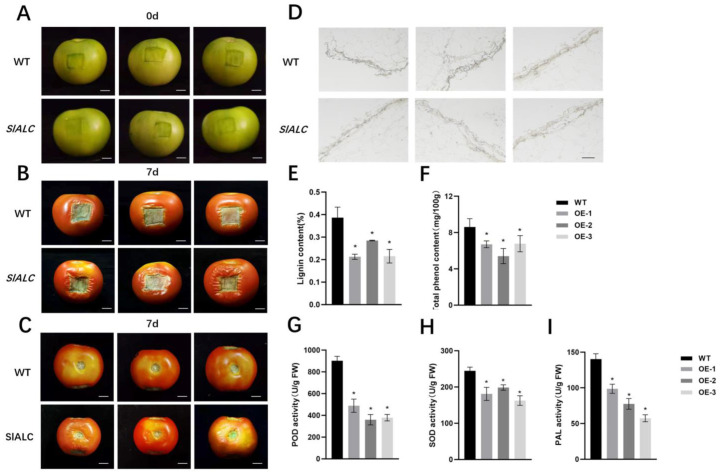
*SlALC*-OE fruits are less lignified compared with wild-type. (**A**–**C**) Comparison of wounds in transgenic and WT fruits. Fruit at 0 days of treatment (**A**). Fruits at 7 days of cultivation (**B**). Fruit shoulders at 7 days of cultivation (**C**). (**D**) Resorcinol staining. (**E**–**I**) Lignin content (**E**). Total phenol content (**F**). POD (**G**), SOD (**H**), and PAL (**I**) activity. Scale bar = 1 cm. Data are means ± SD of three biological replicates. Statistically significant differences were determined using Student’s *t* test (* *p* < 0.05).

*AtALC*, the homologue of *SlALC* in Arabidopsis, has been shown to be involved in regulating the process of siliques dehiscence [31]. In the tomato cv. ’Micro-Tom’, *SlALC* was shown to be involved in the process of fruit lignification [32]. To investigate whether *SlALC* affects fruit dehiscence by influencing fruit lignification, we artificially created the same incisions in the WT as in the *SlALC*-OE lines. After 7 d of incubation, the water loss of the tissues around the *SlALC*-OE fruit wounds was significantly increased compared with the WT, and the peel at the wound was severely wrinkled inwards (Figure 7A–C). Furthermore, wounds of *SlALC*-OE fruits exhibited a decreased content of lignin compared with WT (Figure 7E). Resorcinol staining was used to visualize the lignin content of the tomato pericarp, and the *SlALC*-OE fruits showed a lighter staining than the WT, indicating a lower lignin content (Figure 7D). In addition, the transgenic lines displayed a lower content of polyphenols, the products in the phenylpropane metabolic pathway, and an indicator of lignification compared with WT (Figure 7F). These results indicated that overexpression of *SlALC* decreases fruit lignification and thus leads to fruit cracking more easily.

In addition, antioxidant enzymes have been demonstrated to play an essential role in the lignification of plant tissues, mediating changes in the mechanical properties and stiffness of the exocarp cell wall [32]. Suberin consists of two classes of substances: polyphenols (SPP) and polyaliphatics (SPA). Phenylalanine ammonia-lyase *(*PAL) is a key enzyme in the formation of multiple phenolic monomers in SPP [33]. PAL participates in the phenolic acids produced by the phenylpropane metabolic pathway (a major component of SPP) and is also a key enzyme in the regulation of suberin accumulation [34,35]. Therefore, the activities of the two oxidative enzymes (POD, SOD) and of PAL were examined in tomato wounds. The results showed that the activities of the three enzymes were significantly decreased in the *SlALC*-OE fruits (Figure 7G–I). Furthermore, we used FY staining to observe the suberin of fruits. The results showed that the suberin content of *SlALC*-OE fruits was significantly lower than that of the WT (Figure S1). Our experimental results suggested that *SlALC* may regulate fruit lignification and suberization by influencing SOD, POD, and PAL enzyme activities.

### 2.6. Overexpression of SlALC Affects the Expression of Genes Related to Lignin Synthesis in Fruits

**Figure 8:**
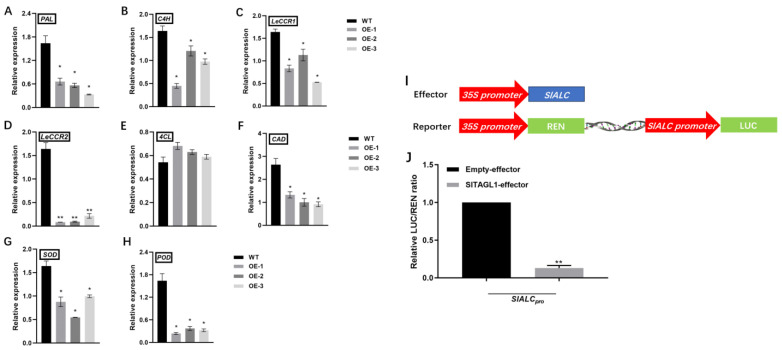
*SlALC* is involved in the regulation of fruit lignification. (**A**–**H**) qRT-PCR analysis of the expression levels of *PAL*, *C4H*, *LeCCR1*, *LeCCR2*, *4CL*, *CAD*, *SOD*, and *POD* in fruit wounds of WT and *SlALC*-OE. (**I**) Effector and reporter constructs used for dual-luciferase assay. (**J**) SlTAGL1 activates *SlALC* promoter by dual-luciferase assay. Data are means ± SD of three biological replicates. Statistically significant differences were determined using Student’s *t* test (* *p* < 0.05; ** *p* < 0.01).

In the phenylpropane metabolic pathway, phenylalanine is catalyzed by PAL to produce cinnamic acid, which forms coumaric acid in the presence of C4H, followed by p-coumaric coenzyme CoA in the presence of 4CL, which is catalyzed by LeCCR and CAD to produce the end product lignin. The expression of these genes was detected in fruit wounds of both WT and *SlALC*-OE lines. The results showed that the expression of *PAL*, a key gene in the phenylpropane metabolic pathway, was down-regulated (Figure 8A), the expression of *C4H* in the shared pathway of lignin and phenolics synthesis was down-regulated (Figure 8B), the expression of *CCR1*, *CCR2*, and *CAD* in the lignin metabolic pathway was significantly decreased (Figure 8C–E), and there was no significant change in the expression of *4CL* (Figure 8E). These results suggested that *SlALC* may regulate the lignification process of fruit by regulating the expression of *PAL*, *C4H*, *CCR1*, *CCR2*, and *CAD*. 

In addition, because of the decreased activity of SOD and POD enzymes (Figure 7G and H), we examined the transcript levels of *SOD* and *POD*. The results showed that the expression of both enzyme genes was downregulated in the fruits of the *SlALC*-OE lines compared with the WT (Figure 8G,H). This is consistent with the results of the enzyme activity assay and suggests that *SlALC* may regulate fruit lignification by modulating the expression of peroxidase genes.

### 2.7. Tomato SlTAGL1 Represses the Activity of the Promoter of SlALC Gene 

The *SHP1* and *SHP2* genes are upstream genes that regulate *ALC* expression in Arabidopsis. An *SHP* homolog, *SlTAGL1*, exists in tomato, and fruits of SlTAGL1-RNAi exhibit increased firmness and lignin synthesis. Therefore, we hypothesized that SlTAGL1 might regulate *SlALC* expression to affect fruit lignification in tomatoes. In order to verify this hypothesis, we constructed a TAGL1-pGreenII 62-SK vector and an SlALC-pGreenII0800-luc vector for dual luciferase reporter experiments (Figure 8I). The results showed that SlTAGL1 could inhibit the activity of the promoter of *SlALC* (Figure 8J), suggesting that *SlTAGL1*, a homologous gene of *SHP* in tomato, may affect fruit lignin synthesis by down-regulating the expression of *SlALC*.

## 3. Discussion

### 3.1. Tomato SlALC Gene Regulates Plant Drought and Salt Tolerance

The involvement of bHLH transcription factors in the regulation of plant stress tolerance has been reported in tomatoes. For example, overexpression of *SlbHLH96* in tomato increased the drought tolerance of plants by stimulating the expression of genes encoding antioxidants and stress-related genes [36]. It has been shown that the bHLH family of PIF transcription factors is associated with stress response. Overexpression of *ZmPIF3.1* and *ZmPIF3.2* genes in rice showed increased drought tolerance by inducing stomatal closure [37]. The *SlALC*-OE lines developed in this study showed a higher rate of seed germination than the WT in a stress environment (Figure 2A–E). Previous studies have shown that *SPT*, a gene homologous to *SlALC* in *Arabidopsis*, is involved in the regulation of seed germination under red light. Lines overexpressing *SPT* had a lower number of dormant seeds during seed germination than the WT [38]. This suggests that *SlALC* may be involved in the seed germination process and enhance seed germination under stress conditions.

Environmental stress usually causes a number of physiological injuries in plants. In this study, *SlALC*-OE plants showed better growth than the WT under drought and salt stress (Figure 3A). Relative water content (RWC) usually reflects the degree of plant damage caused by drought stress [39,40]. In the present study, *SlALC*-OE lines subjected to drought and salt stress had a significantly higher RWC (Figure 3C) and a significantly slower rate of water loss than WT (Figure 3B). In response to drought stress, plants usually accumulate a large amount of proline to increase the water content of the plant as well as the water holding capacity. Proline is a compatible osmotic agent that counteracts drought and salt stress while participating in cellular redox regulation to maintain cellular stability [41,42]. In this study, the ability to accumulate proline was significantly higher in the *SlALC*-OE lines than in the WT under stresses (Figure 3E). The expression of *P5CS*, a key gene controlling proline synthesis, was up-regulated in the *SlALC*-OE lines (Figure 5F). 

Chlorophyll is highly sensitive to stress, and these stimuli alter the total chlorophyll content in the leaf, resulting in a stress response [43]. In the present study, the chlorophyll content of the *SlALC*-OE lines was significantly higher than that of the WT (Figure 3F), indicating an increase in the photosynthetic capacity of the plant. Whereas stronger photosynthesis resulted in increased sugar and starch content in the *SlALC*-OE lines (Figure 3G,I). Consistent with this, the expression of genes related to chlorophyll synthesis (Figure 5L–O), starch synthesis (Figure 5I), and sugar synthesis (Figure 5J) was significantly higher in the *SlALC*-OE lines than in the WT.

Peroxidation of lipid membranes in plants under stress leads to the accumulation of malondialdehyde, which denatures membrane proteins and results in reduced membrane fluidity [44]. Stress usually activates the expression of relevant genes and antioxidant systems to reduce oxidative damage in plants under stress conditions [45]. In this study, there was less accumulation of MDA in *SlALC*-OE lines compared with WT (Figure 4B). The lower conductance of *SlALC*-OE lines compared with WT is related to its relatively intact cell membrane structure, which is able to develop a more stable osmotic pressure. The *SlALC*-OE lines also accumulated less H_2_O_2_ and O_2_**^−^** (Figure 4D,E,G), which may be caused by the increased activity of antioxidant enzymes (Figure 4F,H,I) in the *SlALC*-OE lines as well as the upregulation of genes related to antioxidant enzymes compared with the WT (Figure 5G,H). In conclusion, our experimental results consistently show that the *SlALC* gene is positively involved in the response to drought and salt stress in tomatoes.

### 3.2. SlALC Affects Fruit Lignification, Thereby Influencing Fruit Dehiscence

The *ALC/SPT* gene inhibits fruit lignification both temporally and spatially during fruit development in Solanaceae [46]. It is suggested that *ALC* may affect fruit dehiscence by regulating the process of fruit lignification and that this process is conserved among species. There are many factors affecting fruit dehiscence, and in this study, the *SlALC*-OE fruits were found to be more susceptible to cracking after the rain than the WT (Figure 6A–C). Lignin is an important indicator of the degree of lignification, and it was observed that the lignin content at the wound of *SlALC*-OE fruits was significantly lower than that of the WT (Figure 7D,E). The lignin synthesis pathway is divided into two pathways, one for lignin synthesis and the other for the phenolic pathway to redirect the lignin pathway. Our experimental results showed that the lignin synthesis pathway was inhibited in both *PAL* transcript level (Figure 8A) and its enzyme activity (Figure 7I), followed by a decrease in the expression of *C4H*, *LeCCR*, and *CAD*, which led to a decrease in the final fruit lignin content (Figure 7B–F). In the second part of the pathway, SOD and POD enzyme activities (Figure 7G,H) and gene expression (Figure 8G,H) were reduced. These peroxidases are involved in the polymerization of phenolics to form lignin, and the reduction of their transcriptional activities as well as enzyme activities may lead to the blockage of the oxidation reaction process in this pathway, so that lignin precursors free in the cytoplasm are unable to aggregate in the cell wall to form lignin, ultimately reducing the lignin content of fruits [32]. The experimental results suggest that *SlALC* may act as a negative regulator of lignin synthesis to influence fruit lignification. Reduced lignification resulted in weaker pericarp of tomato and weaker mechanical properties of the pericarp cell walls, all of which indicate that fruits are more susceptible to cracking when subjected to external forces.

*Arabidopsis SHP1/2* has small fruits with excessively lignified valves, and SHP can positively activate *ALC* expression to regulate lignification in *Arabidopsis* [47]. In tomato, the homologue of the *Arabidopsis SHP* gene is *SlTAGL1*, and the dual-luciferase reporter assay revealed that SlTAGL1 could repress the activity of the promoter of *SlALC* (Figure 8I). However, the lignin content of *SlTAGL1*-RNAi fruits increased [48], a process that should be accompanied by a deregulation of the repression of *SlALC* expression, which seems to contradict our observation that the *SlALC*-OE lignin content was reduced. The fact that plants have a large gene regulatory network suggests that there may be other regulatory pathways to affect tomato fruit lignification in *SlTAGL1*-RNAi fruits or *SlALC*-OE fruits, which will need to be verified in further experiments.

In conclusion, we provide new insights into the biological functions of the *bHLH* member *SlALC* in the regulation of drought and salt tolerance, and the phenotypes observed in transgenic plants are expected to be used to improve drought tolerance in crops. Meanwhile, *SlALC* is involved in the regulation of fruit lignification and affects fruit dehiscence, providing new ideas for improving tomato fruit quality.

## 4. Materials and Methods

### 4.1. Plant Material Stress Treatments

Wide-type (*Solanum lycopersicum* L. cv. ‘Ailsa Craig’), *Nicotiana benthamiana*, and *ALC*-overexpression plants were grown in a greenhouse under long-day conditions (16 h of light in 26 °C; 8 h of darkness in 20 °C). For tissue expression analysis, the following samples were collected from WT tomato plants: roots (RT), stems (ST), young leaves (YL), mature leaves (ML), senescent leaves (SL), sepals (SE), flowers (FL), and fruits at the immature green (IMG) (about 25 days after flowering), mature green (MG) (about 30 days after flowering), breaker (B), B + 4, and B + 7 stages.

In drought stress, tomato plants with roots were removed from the soil and then gently washed with water to remove the soil from the roots and fully expose the roots, after which the plants were placed on dry filter paper and incubated at 25 °C for 1 h, 2 h, 4 h, 8 h, 12 h, 24 h, and 48 h. To analyze the expression pattern under salt stress, the roots of tomato plants were irrigated with 300 mM NaCl and incubated for 1 h, 2 h, 4 h, 8 h, 12 h, 24 h, and 48 h. All these samples were immediately wrapped in foil and frozen and stored in a −80 °C freezer.

### 4.2. Sequence Analysis and Phylogenetic Tree Construction of SlALC 

The homologous sequences of SlALC proteins in tomato, *Arabidopsis thaliana*, chili pepper, potato, petunia, and tobacco were obtained from the NCBI (Bethesda, MD, USA) online database and analyzed by multiple sequence comparison using DNAMAN. Phylogenetic tree analysis of homologous proteins was performed using MEGAX (Philadelphia, Pennsylvania, USA).

### 4.3. Subcellular Localization Experiment

The full-length sequence of the open reading frame of the *SlALC* was ligated into the pBI121 vector to generate a 35S::*SlALC*-GFP fusion expression vector and transferred into *Agrobacterium tumefaciens* strain GV3101. The recombinant Agrobacterium was injected into tobacco leaves to allow transient expression of 35S::*SlALC*-GFP in tobacco leaves. After 72 h of co-culture, the fluorescence signals of the samples were observed under a Leica TCS SP8 confocal laser scanning microscope. All primer sequences used in this experiment are listed in Table S1.

### 4.4. Seed Germination and Seedling Growth Assay under D-Mannitol and Salt Treatment 

The wild-type and *SlALC*-OE seeds were sterilized and germinated in a shaker for two days. The germinated seeds were sown into sterile medium containing 0 mM, 50 mM, 100 mM, and 150 mM mannitol and 0 mM, 40 mM, 80 mM NaCl, respectively, for 21 d of constant dark incubation. Another group of germinated seeds were sown into medium with 0 mM, 75 mM, 150 mM, and 300 mM mannitol, and the lengths of hypocotyls and roots were measured after 9 d of incubation (three biological replicates). 

### 4.5. Drought and Salinity Stress Tolerance Experiment

Five-week-old WT and *SlALC*-OE plants were selected, watered fully to allow the plants to completely absorb water, and then treated without watering for 21 days. The specific method of salt treatment was as follows: WT and transgenic tomato plants with same growth size were watered with 100 mL of 300 mM NaCl every 3 d for 15 days. Photographs were taken to document the growth of WT and *SlALC*-OE lines after treatment. The leaves located beneath the plants’ stem tip after treatment were harvested and stored at −80 °C for subsequent determination of relevant physiological indexes [49].

### 4.6. Measurement of Physiological Parameters 

For the water loss rate determination, leaves of WT and *SlALC*-OE lines were weighed immediately and recorded as fresh weight (FW), weighed at 1-h intervals over a 24-h period, and the last recorded W0. Water loss was calculated using the following formula: Water loss (%) = 100% × (FW W0)/FW. 

The proline content was determined by first grinding the leaf material in liquid nitrogen. Then, 0.2 g of this ground material was mixed with 1.3 mL of 3% sulfosalicylic acid and heated in a boiling water bath for 10 min. After cooling to room temperature, the mixture was centrifuged at 12,000 rpm for 15 min. To 1 mL of the supernatant, 1 mL of glacial acetic acid and 1 mL of acid ninhydrin were added in a 10-mL centrifuge tube. This mixture was then heated in a boiling water bath for 30 min. After cooling, 3 mL of toluene was added, and the solution was mixed by inversion and allowed to stand for 10 min before measuring the absorbance at 520 nm (OD_520_). The proline content was calculated using the following formula: The proline content (µg/g) = (C × Vt) / (W × Vs) × 100%, where C is the proline concentration (µg/mL) from the standard curve, Vt is the total volume of the sample extract (mL), W is the fresh weight of the leaf (g), and Vs is the volume of the sample extract used in determining absorbance (mL) [50].

For the determination of soluble proteins as well as enzyme activities, the collected leaves were added to pre-cooled 1.8 mL phosphate buffer solution, ground to homogeneity in an ice bath, and centrifuged at 16,000× *g* at 4 °C for 20 min, and the supernatant was used for the determination. 

Determination of soluble protein content: The samples were mixed with a BSA standard solution, water, and Coomassie Brilliant Blue, and the absorbance was measured at OD_595_. A standard curve was then plotted. Soluble protein content was calculated using the formula: Soluble protein content (mg/g) = (C × Vt)/(W × Vs) × 100%**.** Where C is the soluble protein content (mg/g) in the sample tube from the standard curve; Vt is the total volume of the reaction solution (mL); W is the mass of the ground leaf (g); Vs is the volume of the enzyme solution to be tested (mL) [51]. 

Determination of CAT activity: The samples were mixed with H_2_O_2_, H_2_O, and enzyme solution, and then the rate of decrease in OD at 240 nm was rapidly measured. CAT activity (µg/min) was calculated using the formula: (ΔA_240_ × Vt)/(W ×Vs ×0.01×t), where ΔA_240_ represents the change in A_240_ per unit of time. The meanings of Vt, Vs, W, and t are the same as those used to determine POD activity [52].

Determination of SOD activity: Samples were mixed with PBS, 220 mmol/L Met, NBT, riboflavin, and enzyme solutions. Four control groups were established where the enzyme solution was substituted with a buffer solution. One control group was kept in darkness, while the remaining three controls and the experimental group were exposed to fluorescent light. The dark control group served as a reference for measuring A_560_. SOD activity (μg/FW) was calculated using the formula: SOD activity (μg/FW) = [(Ack − Ae) × V]/(1/2 × W × Vt), where Ack represents the average A_560_ of the three blank groups under light, and Ae represents the A_560_ of the experimental group under light. Vt is the total reaction volume (milliliters); V is the volume of enzyme solution tested (milliliters); W is the fresh weight (grams); t is the reaction time (minutes) [53].

Determination of POD activity: Samples were mixed with H_2_O_2_, guaiacol, and PBS, then added 0.05 mL of enzyme solution to initiate the reaction. At 470 nm, the increase in OD with time was noted. The formula to calculate POD activity (μg/min) is POD activity (μg/min) = (ΔA_470_ * Vt)/(W × Vs × 0.01 ×× t), where ΔA_470_ is the change in A_470_ over a unit time interval; Vt is the total reaction volume (milliliters); Vs is the volume of enzyme solution tested (milliliters); W is the fresh weight (grams); t is the reaction time (minutes) [54].

Determination of relative water content (RWC): The dry weight of the leaves was measured. Relative plant water content using the following formula: RWC (%) = [(WF − WD)/(WS − WD)] × 100%. WF is the weight of the blade at the time of sampling; WD is the weight of the blade after drying; WS is the weight of the blade after complete water absorption and expansion.

Reducing sugar content was determined using Ferring’s reagent. Reducing sugar content was calculated using the following formula: Reducing sugar content (μM/g) = (C × Vt)/(W × Vs). C is the glucose content obtained from the standard curve, and Vs is the volume of extracted liquid used in the determination of A_590_. 

Starch content was determined using perchloric acid. Starch content was quantified using the formula: starch content (μM/g) = (0.9 × C × Vt × n)/(W × Vs × 1000) × 100%. The coefficient 0.9 was used to convert the measured glucose into its starch equivalent. The other parameters used in this formula remained consistent with those used in the reducing sugar content calculation [55].

Chlorophyll content was mainly determined by ethanol extraction method. To determine the total chlorophyll content in mg/mL, the following formula was used: (20.29 × A_646_ + 8.02 × A_663_) × extracted liquid volume (mL)/fresh weight of material (g) [55].

To determine the relative conductivity of the leaves, the leaves of WT and *SlALC*-OE plants were perforated using a perforator. Thirty leaves were taken from each group and immersed in 30 mL of ddH_2_O for 12 h. The conductivity R1 was determined by a conductivity meter, and the conductivity R2 was determined after cooling in a boiling water bath for 30 min. Relative conductivity = (R1/R2) × 100%. 

To determine the MDA content in µmol/L, the following formula was applied: MDA content (µmol/L) = [6.45 × (OD_532_− OD_600_) − 0.56 × OD_450_] × Vt/(Vs × W). Here, OD_532_ and OD_600_ refer to the optical densities at 532 nm and 600 nm, respectively, whereas OD_450_ is the optical density at 450 nm. Vt represents the total volume of the tomato sample extract in milliliters, Vs is the volume of the extract used during the measurement, and W is the net weight of the sample in grams [56].

The H_2_O_2_ content was measured in µM/g using the following calculation formula: = (C × Vt)/(W × Vs) × 100%. Here, C denotes the H_2_O_2_ concentration in µM/L as determined from the standard curve; Vt is the total volume of the sample extract in milliliters; W is the fresh weight of the leaves in grams; and Vs is the volume of sample extract used in the determination of absorbance [56].

### 4.7. DAB, NBT, and Trypan Blue Staining Methods

H₂O₂ reacts rapidly with DAB to form brown compounds catalyzed by peroxidase, thus locating H₂O₂ in plant tissues [57]. The collected samples were immersed in DAB staining solution (1 mg/mL pH3.8) for 8 h. Ethanol (95%) was added to decolorize the samples. To visualize the O_2_− using NBT, tomato leaves were placed in the NBT dye solution so that the leaves were completely infiltrated with the dye. The tubes were placed on a shaker at 27 °C and 100 rpm for 4 h; after 4 h, the color was decolorized by adding ethanol [58]. 

For trypan blue staining, the leaves were placed in 0.1% trypan blue dye solution, treated at 95 °C for 10 min, and left in the dark at room temperature for 30 min. After dyeing, the leaves were decolorized by placing them in 95% ethanol [59].

### 4.8. Construction of SlALC Overexpression Vector and Plant Transformation

The WT tomato cDNA was used as a template, and the full-length coding region of *SlALC* was amplified using specific primers (Table S1) and inserted into the plant overexpression vector pBI121, then the recombinant plasmid was introduced into *Agrobacterium* strain LBA4404. Finally, the recombinant Agrobacterium was transformed into WT tomato to acquire transgenic plants through the infection of tomato cotyledons [60]. 

### 4.9. RNA Extraction and Quantitative Real-Time PCR Analysis

According to the instruction manual, total plant RNA is extracted using RNAiso plus (Takara), and then the M-MLV Reverse Transcriptase kit (Promega, Beijing, China) is used to reverse transcribe the RNA to cDNA. Detailed steps are based on previous research [61]. CFX Connect Real-Time System, transcript levels of specific genes were quantified using SYBR Premix Ex Taq II kit (Takara) and gene-specific primers. The *SlCAC* gene was used as internal control [62]. The complete experimental methodology was conducted according to a previous report [49]. Table S1 lists the primers used in the reverse transcription and qPCR.

### 4.10. Dual-Luciferase Assay

The coding sequence of *SlTAGL1* was amplified and ligated into pGreenII62-SK vector as effector, and the promoter fragment of *SlALC* was inserted into pGreenII 0800-LUC vector as reporter, respectively. Firefly luciferase (LUC) and rabbit luciferase (REN) activities were determined as described in previous study [61]. Primers are listed in Table S1.

### 4.11. Fruit Dehiscence Rate and FY Staining Method

After dehiscence occurred in WT and *SlALC*-OE fruits, the number of cracked fruits (D1) was counted in 100 fruits each, and the fruit dehiscence rate was calculated = D1/100 * 100%.

Samples of wounded fruits were taken, tissue sections were made, dewaxed and rehydrated, FY stained, in aniline blue stained, sealed, and the results were observed by fluorescence microscopy.

### 4.12. Determination of Lignin and Phenolics Content

To determine the lignin content, the fruits were ground, and the samples were mixed with 5 mL of 25% bromoacetyl-acetic acid solution and 0.2 mL of perchloric acid in a constant-temperature water bath at 80 °C for 40 min. After cooling, the reaction was terminated by adding 10 mL of a 2 mol/L NaOH solution and 5 mL of glacial acetic acid. Centrifugation was performed for 10 min, and the supernatant was collected by adding 980 uL of glacial acetic acid, and the absorbance was measured at A_280_. Lignin content was calculated using the following formula: Lignin content (%) = (V × A_280_)/(W × C) × 100% [48]. 

The samples were mixed with 750 μL of Folin-Ciocalteu reagent and 600 μL of sodium carbonate and incubated at 50 °C for 10 min, and the absorbance was measured at 765 nm. The total phenolic content was calculated using the following formula: Total phenolic content (mg/g) = (V × C)/(W × Vs) × 100%. Where V is the volume of sample extract (mL); C is the phenolic content obtained from the standard curve (mg/g); W is the weight of the sample (g); Vs is the volume of the sample at the time of determination (mL) [63]. 

### 4.13. Statistical Analysis

Statistical analysis was performed using Student’s t test. Data were expressed as mean ± SD (standard deviation). Data were analyzed using SPSS 26.0 software. All measurements were taken from the mean of at least three independent biological replicates.

## 5. Conclusions

In this study, a *bHLH* gene, *SlALC*, was isolated, and it was found that overexpression of *SlALC* enhances drought and salt tolerance in tomato plants. Various stress-related physiological indicators also demonstrated the tolerance of *SlALC*-OE plants. Furthermore, *SlALC*-OE fruits were more susceptible to dehiscence after rainfall, and fruit dehiscence-related indicators such as water content, lignin content, and oxidative enzyme activity showed a decreasing trend, while the expression of genes related to fruit lignification was also consistently downregulated. Subsequent investigations revealed that tomato *SlTAGL1* has the ability to exert a negative influence on the promoter activity of *SlALC*. This indicates that SlTAGL1 impacts the process of fruit dehiscence by controlling the mRNA level of *SlALC*. In conclusion, this study provided new insights into the biological functions of the *bHLH* member *SlALC* in the regulation of drought and salt tolerance, and the phenotypes observed in transgenic plants are expected to be used to improve drought tolerance in crops. Meanwhile, *SlALC* is involved in the regulation of fruit lignification and affects fruit dehiscence, providing new ideas for improving tomato fruit quality. The present study may pave the way for more rational selection of tomato fruit traits in molecular breeding, thereby contributing to the development of higher-quality horticultural crops.

## Data Availability

Data are contained within the article or Supplementary Materials.

## Supplementary Materials

The following supporting information can be downloaded at: https://www.mdpi.com/article/10.3390/ijms25179433/s1.
