# Supplementary figures and images for: Overexpression of SlALC Increases Drought and Salt Tolerance and Affects Fruit Dehiscence in Tomatoes
Source: Int J Mol Sci. 2024 Aug 30;25(17):9433. doi: 10.3390/ijms25179433 (PMC11395450; doi:10.3390/ijms25179433)

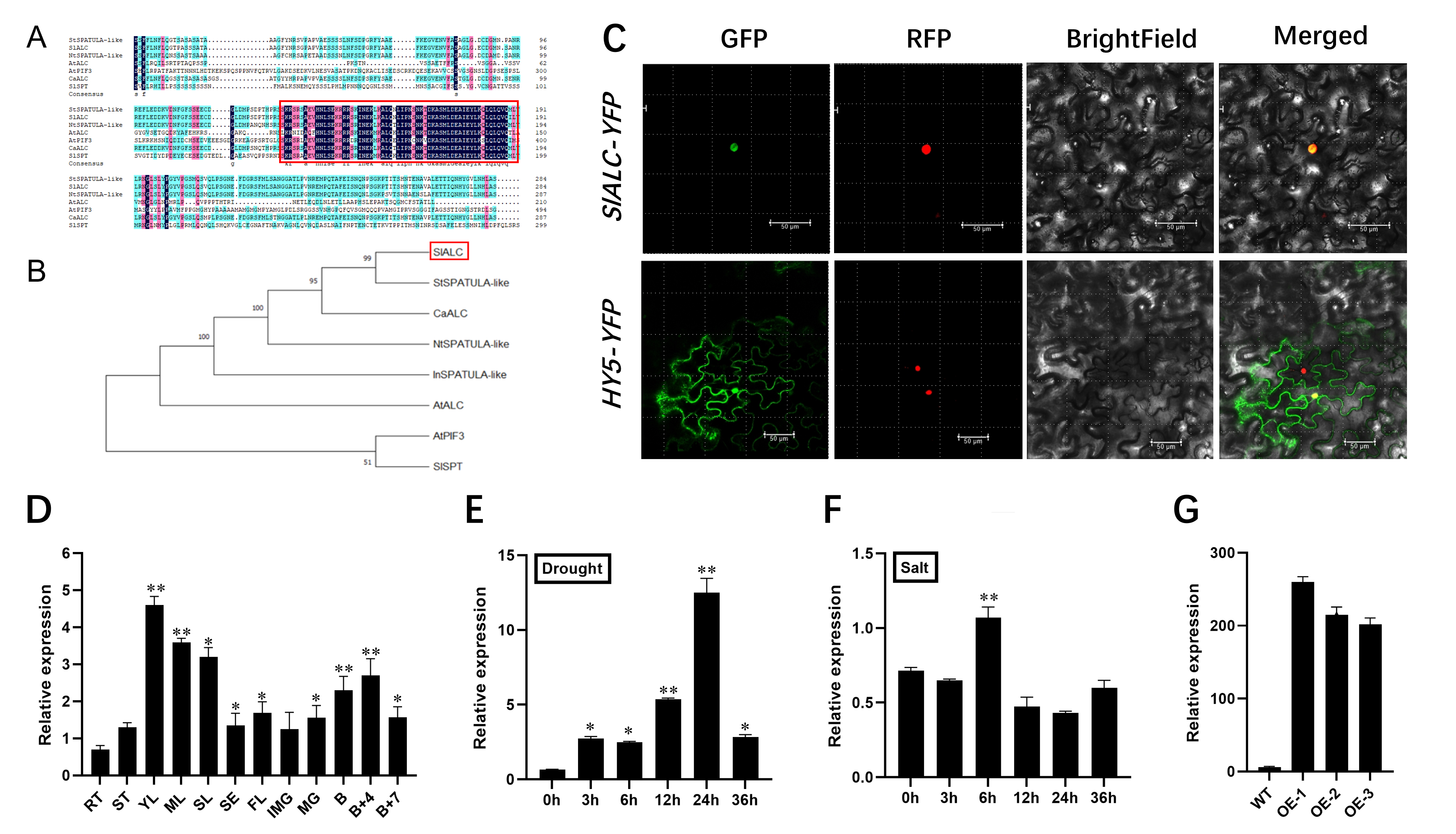

Supplement: Supplementary file 1 [file ijms-25-09433-s001.zip › figure/F1.jpg]

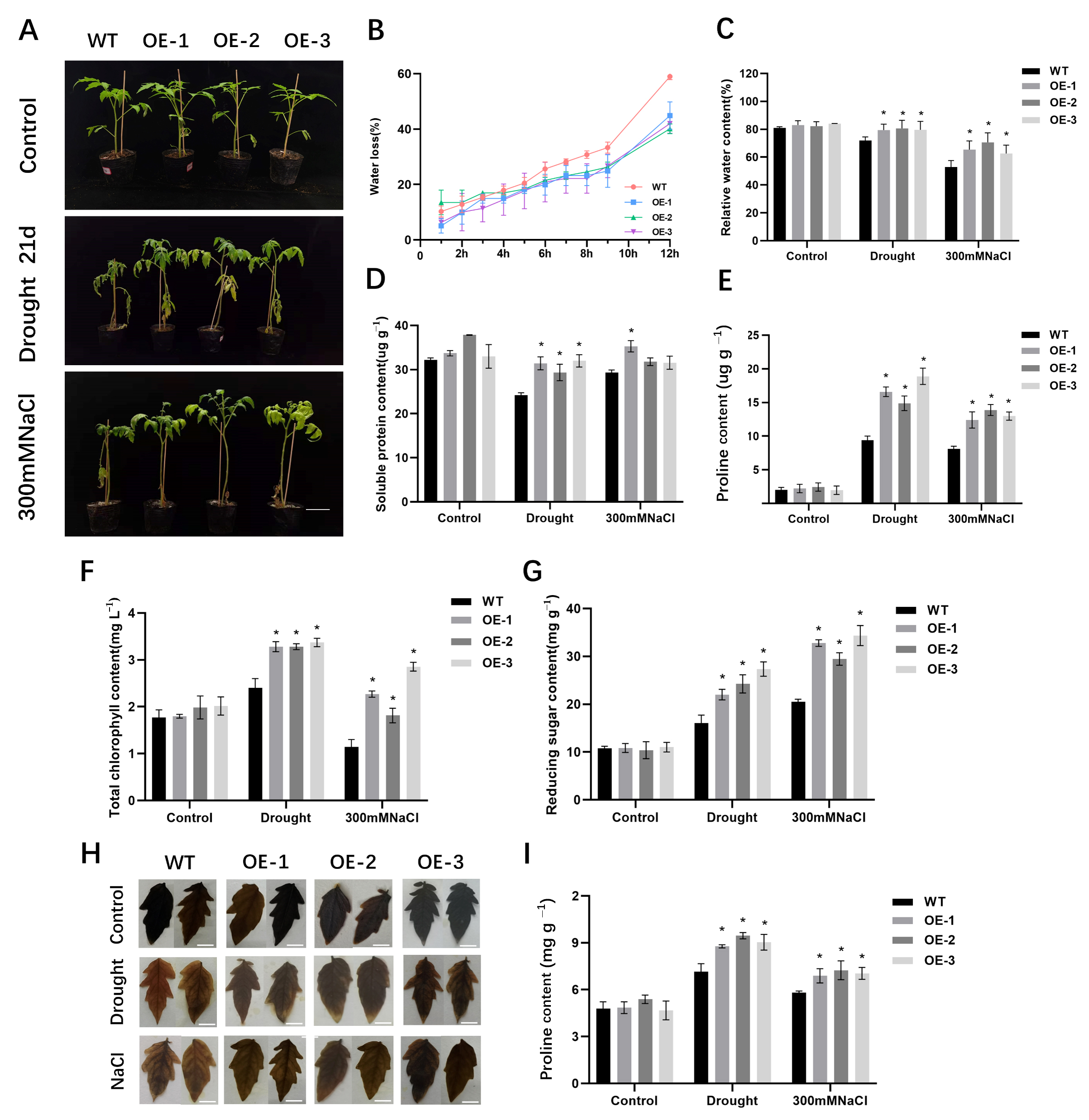

Supplement: Supplementary file 1 [file ijms-25-09433-s001.zip › figure/F3.jpg]

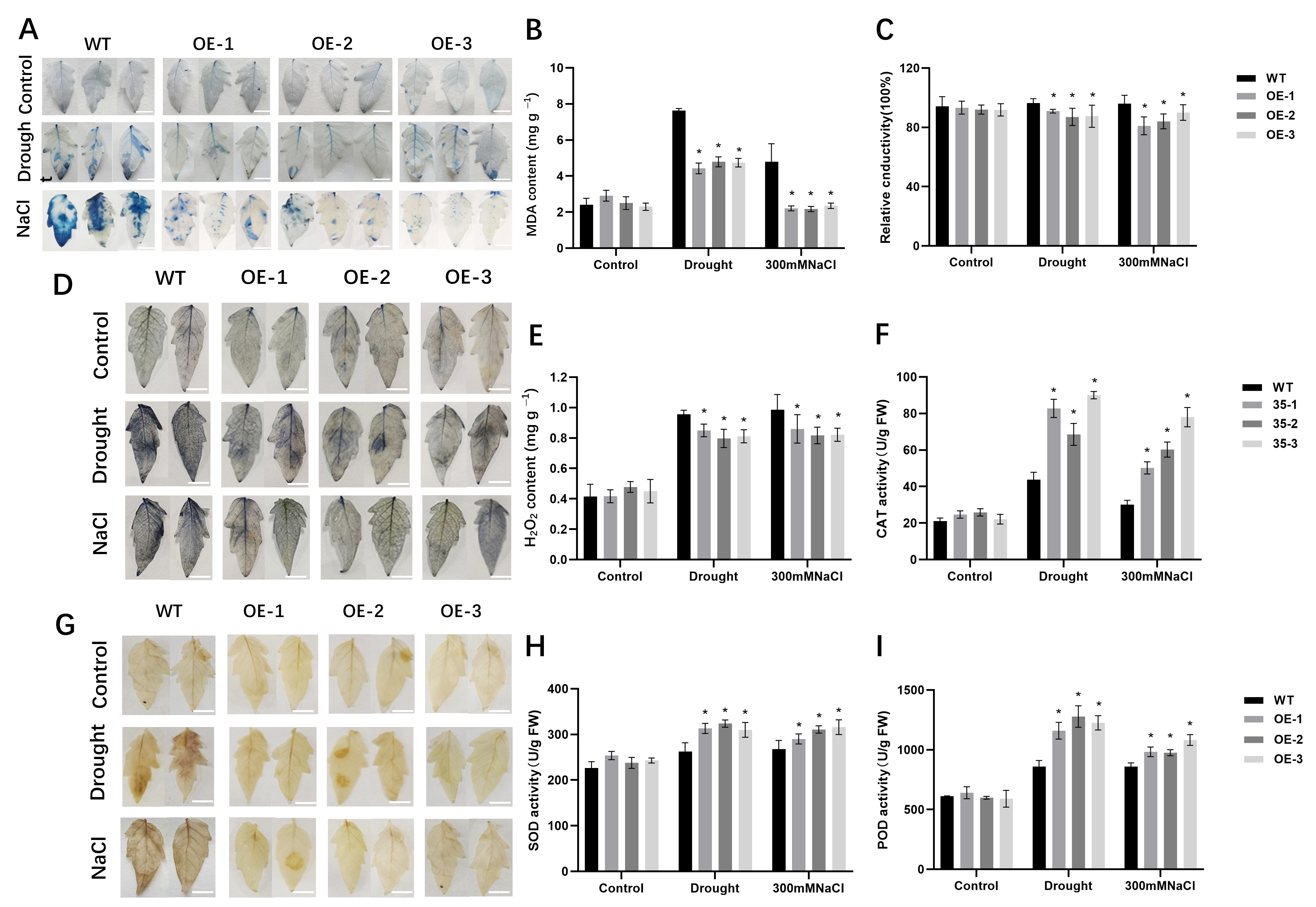

Supplement: Supplementary file 1 [file ijms-25-09433-s001.zip › figure/F4.jpg]
